# Functional Significance of Human Resting-State Networks Hubs Identified Using MEG During the Transition From Childhood to Adulthood

**DOI:** 10.3389/fneur.2022.814940

**Published:** 2022-06-23

**Authors:** Sheraz Khan, Javeria Ali Hashmi, Fahimeh Mamashli, Matti S. Hämäläinen, Tal Kenet

**Affiliations:** ^1^Department of Neurology, Massachusetts General Hospital and Harvard Medical School, Boston, MA, United States; ^2^Department of Radiology, Massachusetts General Hospital and Harvard Medical School, Boston, MA, United States; ^3^Athinoula A. Martinos Center for Biomedical Imaging, Massachusetts General Hospital, Charlestown, MA, United States; ^4^Department of Anesthesia, Pain Management, and Perioperative Medicine, Dalhousie University, Halifax, NS, Canada

**Keywords:** development, brain connectivity, rhythms, graph theory, magnetoencephalography

## Abstract

Cortical hubs identified within resting-state networks (RSNs), areas of the cortex that have a higher-than-average number of connections, are known to be critical to typical cognitive functioning and are often implicated in disorders leading to abnormal cognitive functioning. Functionally defined cortical hubs are also known to change with age in the developing, maturing brain, mostly based on studies carried out using fMRI. We have recently used magnetoencephalography (MEG) to study the maturation trajectories of RSNs and their hubs from age 7 to 29 in 131 healthy participants with high temporal resolution. We found that maturation trajectories diverge as a function of the underlying cortical rhythm. Specifically, we found the beta band (13–30 Hz)-mediated RSNs became more locally efficient with maturation, i.e., more organized into clusters and connected with nearby regions, while gamma (31–80 Hz)-mediated RSNs became more globally efficient with maturation, i.e., prioritizing faster signal transmission between distant cortical regions. We also found that different sets of hubs were associated with each of these networks. To better understand the functional significance of this divergence, we wanted to examine the cortical functions associated with the identified hubs that grew or shrunk with maturation within each of these networks. To that end, we analyzed the results of the prior study using Neurosynth, a platform for large-scale, automated synthesis of fMRI data that links brain coordinates with their probabilistically associated terms. By mapping the Neurosynth terms associated with each of these hubs, we found that maturing hubs identified in the gamma band RSNs were more likely to be associated with bottom-up processes while maturing hubs identified in the beta band RSNs were more likely to be associated with top-down functions. The results were consistent with the idea that beta band-mediated networks preferentially support the maturation of top-down processing, while the gamma band-mediated networks preferentially support the maturation of bottom-up processing.

## Introduction

The period from childhood to adolescence is a time window of extensive developmental changes in the neurophysiological topology of the brain (1, 2). This period of rapid growth and reorganization also coincides with a delicate period of increased vulnerability to neuropsychiatric disorders, further underscoring the need to gain insight into the changes that underlie this period. As part of cortical maturation, the distribution of functional connections also changes so that some brain regions acquire a higher-than-average number of connections to form hubs, while other hubs that may have been prominent during childhood may shrink with maturation. Hubs play a key role in integrative processing and supporting connectivity between network modules (3, 4), and are implicated in a range of brain-based disorders (5). To date, the vast majority of studies of cortical changes during maturation have focused on resting-state networks due to their replicability across sites and relevance to a wide range of psychiatric and neurological disorders (6–13). Almost all these studies have been carried out using functional magnetic resonance imaging (fMRI), i.e., with signals that fluctuate in the infra-slow range. Thus, to date, it has not been known whether or how hub maturation patterns vary as a function of the frequency band mediating their connectivity. This question is relevant because intrinsic cortical rhythms are themselves functionally significant, and rhythm-specific alterations emerge are widely reported for a wide range of brain-based disorders and diseases (14–21). Studying the maturing and changing distribution and characteristics of hubs formed by intra-areal synchronization of specific intrinsic brain rhythms is, therefore, necessary for a better understanding of the maturing brain and parsing the functional relevance of developing hubs can offer insights into brain function and underscore sensitive periods underpinning developmental disorders.

We have previously observed that developmental changes in the segregation and integration of resting-state networks and their corresponding hubs are clearly observable within specific cortical rhythms and vary by rhythm (22). Specifically, we showed that there were no notable maturational changes mediated by the slower brain rhythms (delta, 1–3 Hz; theta, 4–7 Hz; alpha, 8–12 Hz). In contrast, the resting state networks mediated by the faster beta (13–30 Hz) and gamma (30–80 Hz) frequency bands undergo marked topological reorganization during maturation between the ages 7 and 29. Networks mediated by the beta brain rhythm become more integrated with maturation, i.e., more organized into clusters, i.e., prioritizing communication between nearby hubs. In contrast, networks mediated by the gamma brain rhythm become more segregated and distributed with maturation, i.e., prioritizing faster signal transmission between distant hubs. As part of that same study, we found that maturation-driven changes in network topology resulted in the hubs expanding (getting more connections) or shrinking (losing connections and potentially losing hub “status”) in resting-state networks mediated by the beta and gamma bands. Spatially, maturing hubs in the gamma band-mediated networks were located in heteromodal regions, such as the posterior parietal cortex, posterior cingulate cortex, and the anterior insula, in agreement with fMRI studies (23, 24). Hubs in the beta-band-mediated network were located in heteromodal-frontal regions and shrunk with maturation, which is a finding hitherto unobserved with fMRI.

In our prior study, we speculated that the altered spatial distribution of hubs in both networks reflects a shift in higher-order cognitive processes and thus top-down processing, within the beta band-mediated networks, and in bottom-up sensory functioning in the gamma band-mediated networks. This hypothesis was derived from recent data on the putative roles of the beta and gamma bands in intra-areal synchronization. It has been demonstrated that intra-areal gamma-band synchronization mediates bottom-up signaling of sensory inputs in several studies (17, 25). Relatedly, top-down influences on sensory processing, such as attentional selection and cognitive control, are mediated by intra-areal, alpha-beta band synchronizations (17, 25, 26). The developmental changes in hubs observed with MEG indicate an increased clustering and segregation in beta and gamma-mediated networks, respectively.

In this study, we investigated these hypotheses. To that end, we conducted a meta-analysis that built on the results and data from the prior paper. Specifically, we used Neurosynth, a meta-analytic platform that relies on a large-scale, automated synthesis of fMRI data for data mining (27) to test and substantiate the interpretations of the results proposed in our prior study. The Neurosynth platform allows association tests for identifying the relevance of a brain region to categories of behavioral functions in a statistically principled manner and has been used successfully in multiple studies to gain an understanding of the potential function of hubs (28–33). The Neurosynth platform can be tapped in two ways. In the “reverse” direction, called “decoding,” the input to Neurosynth is the coordinate of interest, i.e., the coordinate of the hubs, and the output is the terms associated with these coordinates, ranked by the probability of association. We hypothesized that maturing hubs identified in the beta band network will be associated with Neurosynth terms related to top-down processing while maturing hubs identified in the gamma band network will be associated with terms related to bottom-up processing. In the “forward” direction, one enters a brain-related term of interest. As an output, Neurosynth returns the coordinates of the brain areas associated with these terms based on the papers analyzed in its database in probabilistic ranking order. Therefore, the coordinates most often associated with the term depression, for instance, will be ranked at the top of the search results, and so on. We used this approach to test for the extent of overlap between hubs associated with terms related to bottom-up or top-down processing, and the hubs identified in our analyses. We hypothesized that maturing hubs identified in the beta band network will overlap with hubs associated with terms related to top-down processing while maturing hubs identified in the gamma band network will overlap with hubs associated with terms related to bottom-up processing (22).

## Materials and Methods

### Participants

Magnetoencephalography resting-state data were collected from 145 healthy typically developing participants, aged 7–29. Due to excessive motion, data from 14 subjects were discarded, resulting in 131 high-quality datasets (64 females) with a roughly uniform age distribution. Because we combined datasets across several different studies that utilized the MEG at the Martinos Center at the Massachusetts General Hospital, no single behavioral measures were available across all the participants. IQ measured with the Kaufman Brief Intelligence Test – II (34) was available for 68 of the participants. Within this subgroup, no significant change in IQ with age was observed, as expected, given that IQ is normalized by age. All the studies that were pooled for this analysis were screened for typical development and health. All the adult (age 18+) participants signed a consent form, agreeing to participate in the study, and consent forms were signed by the parents of the participants aged 7–17. The participants aged 14–17 were also invited to sign a consent form if they wished to do so. All procedures and forms were approved by the Massachusetts General Hospital IRB.

### Experimental Paradigm

The resting-state paradigm consisted of a fixation cross at the center of the screen, presented for 5 min continuously, while the participants were seated and instructed to fixate on the cross. The fixation stimulus was projected through an opening in the wall onto a back-projection screen placed 100 cm in front of the participant, inside a magnetically shielded room.

### MRI Data Acquisition and Processing

T1-weighted, high-resolution MPRAGE (Magnetization Prepared Rapid Gradient Echo) structural images were acquired on either a 1.5 T or a 3.0-T Siemens Trio whole-body MRI (magnetic resonance) scanner (Siemens Medical Systems) using either 12 channels or a 32 channel head coil. The structural data were preprocessed using FreeSurfer (35, 36). After correcting for topological defects, cortical surfaces were triangulated with dense meshes with ~130,000 vertices in each hemisphere. To expose the sulci in the visualization of cortical data, we used the inflated surfaces computed by FreeSurfer.

### MEG Data Acquisition and Cleaning

Magnetoencephalography data were acquired inside a magnetically shielded room (37) using a whole-head Elekta NeuromagVectorView system composed of 306 sensors arranged in 102 triplets of two orthogonal planar gradiometers and one magnetometer. The signals were filtered between 0.1 Hz and 200 Hz and sampled at 600 Hz. To allow co-registration of the MEG and MRI data, the locations of three fiduciary points (nasion and auricular points) that define a head-based coordinate system, a set of points from the head surface and the locations of the four HPI coils were determined using a Fastrak digitizer (Polhemus Inc., Colchester, VT) integrated with the VectorView system. ECG and horizontal (HEOG) and vertical electrooculogram (VEOG) signals were recorded. The position and orientation of the head with respect to the MEG sensor array were recorded continuously throughout the session with the help of four head position indicator (HPI) coils (38).

We also monitored the continuous head position, and the session was restarted if the excessive head movement was recorded. The session was also restarted if any slouching in the seated position was observed. Pillows, cushions, and blankets were fitted to each individual to address slouching and readjusted as needed. In addition to the human resting-state data, 5 min of data from the empty room was recorded before or after each session for noise estimation purposes.

Following this, the data were spatially filtered using the signal space separation (SSS) method (39, 40) with Elekta NeuromagMaxfilter software to suppress noise generated by sources outside the brain. This procedure also corrects for head motion using the continuous head position data described in the previous section. The heartbeats were identified using in-house MATLAB code modified from the QRS detector in BioSig (41). Subsequently, a signal-space projection (SSP) operator was created separately for magnetometers and gradiometers using the singular value decomposition (SVD) of the concatenated data segments, containing the QRS complexes and separately identified eye blinks (42), using code now implemented into the open-source MNE-Python software (43). Data were also low-pass-filtered at 144 Hz to eliminate the HPI coil excitation signals.

Artifact cleaning was performed as follows: signal spikes where the amplitude was higher than 5σ over the mean were identified and dropped. To remove the effect of microsaccades, horizontal and vertical EOG channels were filtered at a pass-band of 31–80 Hz. The envelope was then calculated for the filtered signals and averaged to get REOG. Peaks exceeding three SDs above the mean calculated over the whole-time course were identified, and the corresponding periods were discarded from subsequent analysis. Lastly, head movement recordings from the HPI coils were used to drop any 1-s blocks where the average head movement exceeded 1.7 mm/s (an empirical threshold). The amount of data lost through cleaning was well below 10% and did not differ significantly with age.

### MEG Data Processing

**Figure 1:**
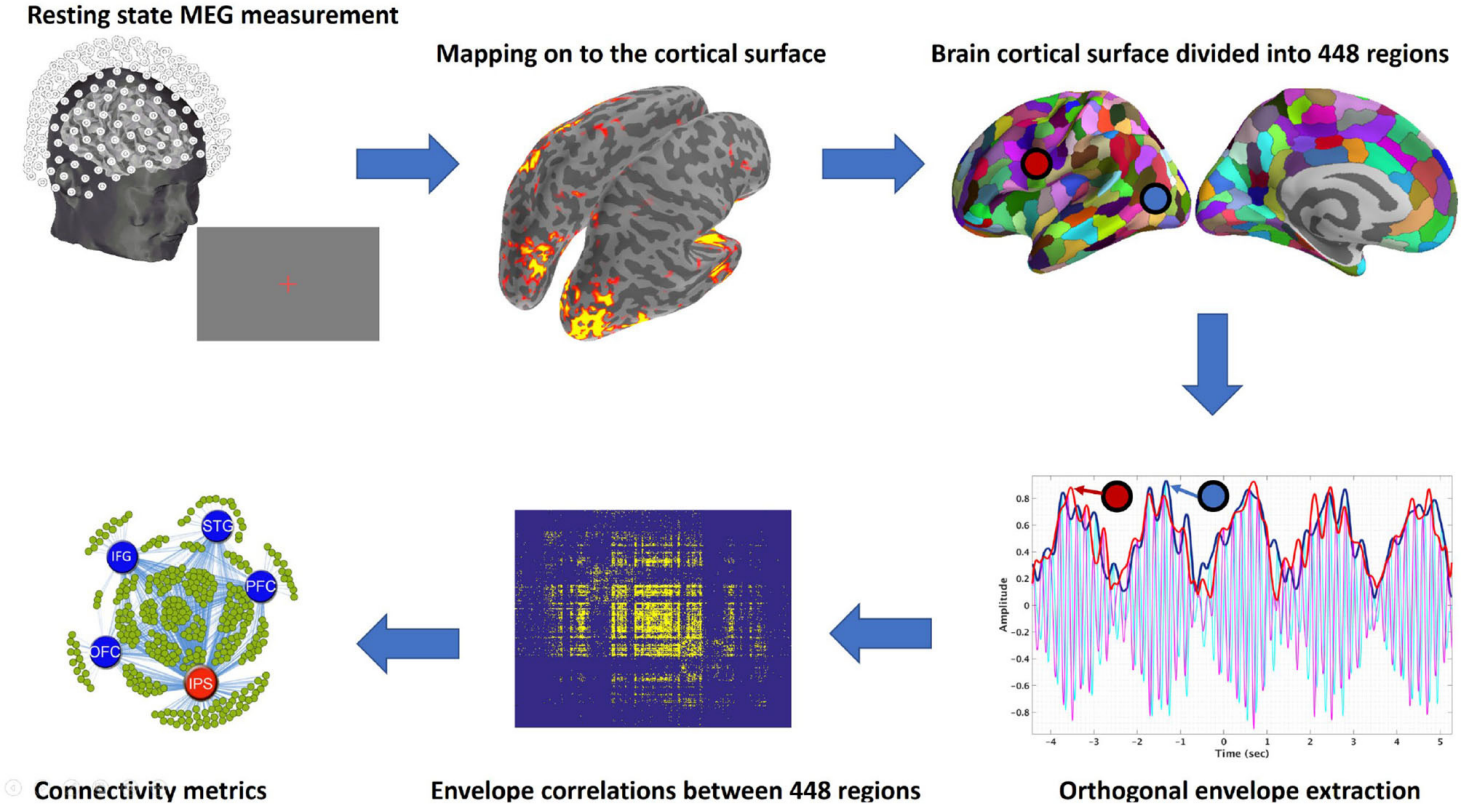
Schematic illustration of pipeline. From top left in a clockwise direction: Resting state data were acquired using MEG, cleaned as described above, and then mapped to the cortical surface. The surface was then divided into regions (parcellated), and envelopes were calculated for each frequency band in each region. The connectivity between the regions was then computed from the envelopes and used to derive the connectivity metrics. Hubs were then identified using betweenness centrality. The whole pipeline from raw MEG data to connectivity metrics is available as an MNE-Python example.

The analysis stream we followed is illustrated in Figure 1, and details are described below.

#### Mapping MEG Data Onto Cortical Space

The dense triangulation of the folded cortical surface provided by FreeSurfer was decimated to a grid of 10,242 dipoles per hemisphere, corresponding to a spacing of ~3 mm between adjacent source locations. To compute the forward solution, a boundary-element model with a single compartment bounded by the inner surface of the skull was assumed (44). The watershed algorithm in FreeSurfer was used to generate the inner skull surface triangulations from the MRI scans of each participant. The current distribution was estimated using the regularized minimum-norm estimate (MNE) by fixing the source orientation to be perpendicular to the cortex. The regularized (regularization = 0.1) noise covariance matrix that was used to calculate the inverse operator was estimated from data acquired in the absence of a subject before each session. This approach has been validated using intracranial measurements (45). To reduce the bias of the MNEs toward superficial currents, we incorporated depth weighting by adjusting the source covariance matrix, which has been shown to increase spatial specificity (46). All forward and inverse calculations were done using MNE-C (47).

#### Cortical Parcellation (Labels)

FreeSurfer was used to automatically divide the cortex into 72 regions (48). After discarding “medial wall” and “corpus callosum,” regions were further divided into a total of *N* = 448 cortical labels so that each label covers a similar area, again using FreeSurfer. This was done to avoid averaging across a large label that crosses multiple sulci and gyri and, therefore, could result in signal cancellation across the label. Lastly, a high-resolution parcellation also reduces the dependence of the results on the specific selection of the parcels.

#### Deriving the Time Series for Each Label

Because of the ambiguity associated with individual vertex (dipole) orientations, the time series for each vertex within a label was not averaged directly but first aligned with the dominant component of the multivariate set of time series before calculating the label mean. To align the sign of the time series across vertices, we used the SVD of the data **X**^*T*^ = **U**Σ**W**^*T*^. The sign of the dot product between the first left singular vector **U** and all other time series in a label was computed. If this sign was negative, we inverted the time series before averaging. The time series were band-pass filtered and downsampled for faster processing, while making sure that the sampling frequency was maintained at *f*_*s*_>3*f*_*hi*_ (obeying the Nyquist theorem and avoiding aliasing artifacts). The chosen frequency bands were delta (1–4 Hz), theta (4–8 Hz), alpha (8–12 Hz), beta (13–30 Hz), and gamma (31–80 Hz). The line frequency at 60 Hz was removed with a notch filter of bandwidth of 1 Hz. Hilbert transform was then performed on these band-pass data. More specifically, for each frequency band, the analytic signal $\hat{X}\left(t\right)$ was calculated by combining the original time series with its Hilbert transform into a complex time series:


$$\begin{array}{l} \hat{X}\left(t\right)=x\left(t\right)+ȷH\left[x\left(t\right)\right] \end{array}$$

The resulting time series $\hat{X}\left(t\right)$ can be seen as a rotating vector in the complex plane whose length corresponds to the envelope of the original time series *x*(*t*) and whose phase grows according to the dominant frequency. Figure 1, Step 4, shows an example of a modulated envelope on the top of the bandpass data (carrier).

#### Deriving the Orthogonal Envelopes

We used envelope correlations to reliably estimate synchronicity between different cortical labels (49). In contrast to phase-based connectivity metrics, envelope correlations measure how the amplitude of an envelope within a frequency band is synchronously modulated over time across distinct cortical regions, as illustrated in the fourth panel of Figure 1. Previous studies (humans and primates) have demonstrated the validity and functional significance of these synchronous envelope amplitude modulations (49–53) for both oscillatory and broadband signals.

To address the field-spread problem associated with MEG data (54), we used the previously described orthogonal (55) variation of the envelope correlation metric. This method requires any two putatively dependent signals to have non-zero lag and is thus insensitive to the zero-lag correlations, stemming from the field spread. Mathematically, the connectivity between two complex signals $\hat{X}$ and Ŷ is calculated by “orthogonalizing” one signal wall-cap concerning the other $Ŷ\left(t,f\right)\to {Ŷ}_{{⊥}^{X}}\left(t,f\right)$, and subsequently taking the Pearson correlation between their envelopes. This is done in both directions, and the two results are averaged to give the final connectivity measure $C_{⊥}\left(\hat{X},Ŷ;t,f\right)$.


$$\begin{array}{l} {Ŷ}_{{⊥}^{X}}\left(t,f\right)=I\left(Ŷ\left(t,f\right)\frac{{\hat{X}}^{†}\left(t,f\right)}{\left|\hat{X}\left(t,f\right)\right|}\right){ê}_{{⊥}^{X}}\left(t,f\right)\\ C_{⊥}\left(\hat{X},Ŷ;t,f\right)=\frac{Corr\left(\left|\hat{X}|,|{Ŷ}_{{⊥}^{X}}\right|\right)+Corr\left(\left|Ŷ|,|{\hat{X}}_{{⊥}^{Y}}\right|\right)}{2} \end{array}$$

Due to the slow time course of these envelopes and to ensure enough independent samples are available in the correlation window (55), we calculated the orthogonal connectivity using an overlapping sliding window of 30 s with a stride of 1/8 of the window size. Note that all 30 epochs that contained a discontinuity due to a noisy segment that had to be removed were excluded from the analyses.

#### Deriving the Connectivity and Adjacency Matrices

As a starting point for calculating the graph-theoretic metrics, we used the connectivity matrix, which contained the orthogonal correlations between all *N* × *N* node pairs and at each time window. A separate matrix was computed for each frequency band. The result of the processing pipeline is a connectivity array of dimension *N* × *N* × *N*_*Time*_ × *N*_*Bands*_ for each subject. To increase the signal to noise, we collapsed the connectivity array along the temporal dimension by taking the median of each pairwise orthogonal correlation across time windows. Thresholding and binarizing the connectivity matrix result in the adjacency matrix *A*.

We used a threshold proportional scheme to retain a given proportion of the strongest connectivity matrix entries in *A*. Specifically, the adjacency matrix *A* was constructed using a fixed cost threshold, ensuring that the density or number of connections of the network is equated across all individuals and age groups. Cost is a measure of the percentage of connections for each label about all connections of the network. Since the total number of connections is the same for all participants and is determined by the number of nodes being considered, the use of a fixed cost, i.e., a fixed percentage threshold, allows for exactly equal numbers of connections across the participants. This is important to ensure graph metrics can be compared across all individuals and age groups. As there was no rationale for using a cost threshold, therefore, we compared graph network properties for a wide range of costs; we used a thresholding range from 5 to 30% at increments of 5%. For the graph metrics to be reliable, they should be consistent over the range of thresholds.

The adjacency matrix *A* defines a graph G in the form of pairs of nodes that are connected by an edge. Thus, *A* is defined such that its binary element *A*_*ij*_ is either 1 or 0, depending on whether the edge *e*_*ij*_ between nodes *v*_*i*_ and *v*_*j*_ exists or not:


$$A_{ij}=\left\{ \begin{array}{l} 1\text{\&}if\;\exists \;e_{ij}\\ 0\&if\;∄\;e_{ij} \end{array}\right.$$

##### Path Length

The average shortest path length between all pairs of nodes was calculated as follows:


$$\begin{array}{l} L=\frac{1}{n\left(n-1\right)}\underset{i\neq j;v_{i},v_{j}\in G}{\sum }d_{ij} \end{array}$$

where the topological distance **d**_**ij**_ between nodes **v**_**i**_ and **v**_**j**_ is defined as the minimum number of edges one must traverse to get from one node to the other


$$d_{ij}=\min\{n|A^{n}\left[i,j\right]\neq 0\}$$

where *A*^*n*^ denotes the *n*th power of the adjacency matrix *A*, and *i* and *j* are row and column indices of the resulting matrix.

##### Degree

The degree (hubness) of a node **v**_**i**_ in a Graph G is defined as


$$\begin{array}{l} D_{i}=\overset{n}{\underset{j=1,j\neq i}{\sum }}e_{ij} \end{array}$$

where *e*_*ij*_ is the *i* th row and *j* th column edge of adjacency matrix *A*.

##### Clustering Coefficient

The local clustering coefficient in the neighborhood of a vertex **v**_**i**_ is defined as the ratio of actual and maximally possible edges in the Graph $G_{\text{i}}$, which is equivalent to the graph density of $G_{\text{i}}$:


$$\begin{array}{l} C_{i}=\frac{2|\left\{e_{jk}\right\}|}{k_{i}\left(k_{i}-1\right)}:v_{j},v_{k}\in G_{i} \end{array}$$

##### Global and Local Efficiencies

Global efficiency measures the efficiency of information transfer through the entire network and is assessed by mean path length. While the concept of path length is intuitive in anatomical networks, it is also relevant for functional networks, since a particular functional connection may travel different anatomical paths, and, while the correspondence between the two is generally high, it is not necessarily identical (56–58). Local efficiency is related to the clustering of a network, i.e., the extent to which nearest neighbors are interconnected. Thus, it assesses the efficiency of connectivity over adjacent brain regions.

The average global efficiency of information transfer in graph G having *n* nodes can be calculated from the inverse of the edge distances *d*_*i, j*_


$$\begin{array}{l} E_{glob}=E\left(G\right)=\frac{1}{n\left(n-1\right)}\underset{i\neq j;v_{i},v_{j}\in G}{\sum }\frac{1}{d_{ij}} \end{array}$$

The quantity above is a measure of the global efficiency of information transfer for the whole graph G. There is also a local efficiency for each vertex *v*_*i*_, measuring how efficiently its neighbors can communicate when a vertex *v*_*i*_ is removed. If the subgraph of all neighbors of *v*_*i*_ is denoted by $G_{i}$, then its local efficiency $E\left(G_{i}\right)$ is approximately equivalent to the clustering coefficient *C*_*i*_ (59).


$$\begin{array}{l} E_{loc}=\frac{1}{n}\underset{v_{i}\in G}{\sum }\;E\left(G_{i}\right) \end{array}$$

##### Betweenness Centrality

Betweenness centrality pertains to individual nodes in the network rather than the network as a whole and assesses how many of the shortest paths between all other node pairs in the network pass through that node. Nodes with high betweenness centrality (hubs) are, therefore, more important for overall network efficiency.

The betweenness centrality of node *i* is defined as


$$\begin{array}{l} b_{i}=\underset{m\neq i\neq n\in G}{\sum }\frac{\sigma_{mn}\left(i\right)}{\sigma_{mn}} \end{array}$$

where σ_*mn*_ is the total number of shortest paths (paths with the shortest path length) from Node *m* to Node *n*, and σ_*mn*_*(i)* is the number of shortest paths from Node *m* to node *N* that pass through Node *i*. Betweenness centrality of a node thus reflects the control and influence of that node on other nodes. Nodes with high betweenness centrality have a high impact on information transferal and collaboration between disparate sub-networks.

### Resilience

Resilience is the graph-theoretic metric most critical to the current analysis and, therefore, merits a more thorough discussion. Resilience measures the robustness of the network if the most heavily connected nodes (hubs) are removed. This measure is inversely related to the capacity of the system for integrating information in an efficient manner and is also reflective of the brain's small-world property, a metric that determines the balance between cost and efficiency proffered by the network for information transfer (60, 61). Small world property and resilience are inversely proportional because both are computed from the relative strength of local and global efficiencies, one directly and one inversely. Indeed, this small world property and resilience for the beta and gamma-mediated networks showed opposite trajectory directions with maturation. We chose this measure because it has been studied, mostly using fMRI, in the context of psychiatric disorders, where multiple hubs might be functioning abnormally (3, 62). It has also been shown that greater resilience in a functionally derived task-driven network is associated with greater inhibitory control cognitively (63), a function that is often impaired in neurodevelopmental and psychiatric disorders. Importantly, the measure incorporates network topology in conjunction with the spatial distribution of hubs, because it takes the degree, i.e., the number of connections, of individual nodes into account.

Resilience quantifies Graph G's robustness to targeted or random attacks. Targeted attacks remove nodes in the descending order of importance (i.e., number of connections). At each attack, global efficiency is computed. Robustness is defined as the ratio of the original efficiency with efficiency calculated after the attack. This process is repeated until a predetermined number of hubs, or all hubs are removed. In this case, to obtain the data shown in **Figures 4**, **5**, we removed the largest 90 hubs (nodes) associated with each term in descending order and computed the relative loss or gain in network efficiency after each removal.

### Bootstrapping and Correlation

To visualize the significance of age effects and assess uncertainties in the graph metrics with respect to age, we used nested bootstrapping with 1,024 realizations. The nested bootstrap procedure approximates the joint distribution of age *x* with the age-dependent network metric *f* (*y*_*x*_), where *f* (*y*_*x*_) is the average network metric over many subjects of age *x* (see notes below). We observed *n* pairs (*x*_*i*_, *y*_*i*_), where *x*_*i*_ is the age and *y*_*i*_ the corresponding imaging data for the *i*^*th*^ subject. Ideally, we would like to observe (x_i_, *Y*_x_), where *y*_x_ denotes the (conceptual) average of subjects chosen at random from a population, where each subject is of age *x*.

Let *f* (*y*) denote the function that maps imaging data to a scalar metric, describing some aspect of a network. Since *y*_*i*_ contains noise, to visualize and estimate uncertainties in graph metrics, we can approximate (*x*_*i*_, ȳ _*x*_) by (${\bar{X}}_{*}$ , ȳ_*_), where the * denotes a bootstrap sample. We can then evaluate *f* (${y_{\bar{x}}}_{*}$) instead of *f* (*y*_*i*_).

Each realization of bootstrapping yielded one average network metric and one value for the mean age of the group. Each data point on the normalized density color map corresponds to one realization of the bootstrap. To evaluate the relationship between network quantity and age, we used Spearman correlation. The *p*-values were computed after correcting for multiple comparisons across the correction space of frequency bands, thresholds, and graph metrics by controlling for a family-wise error rate using maximum statistics through permutation testing (64).

Specifically, the correction for multiple comparisons was done by constructing an empirical null distribution. For this purpose, *n*_*p*_ = 10,000 realizations were computed by first randomizing age and then correlating it with all graph metrics at all thresholds and frequency bands, and finally taking maximum correlation value across this permuted correction space. The corrected *p*-values (*p*_*c*_) were calculated as:


$$\begin{array}{l} p_{c}=\;\frac{2\left(n+1\right)}{n_{p}+1} \end{array}$$

where *n* is the number of values in the empirical null distribution greater or lower than the observed positive or negative correlation value, respectively. The factor of two stems from the fact that the test is two-tailed. Correlations resulting in significant *p*-values were then again tested using Robust Correlation (65), which strictly checks for false-positive correlations using bootstrap resampling.

### LOESS Regression

LOESS, which stands for Locally Estimated Scatterplot Smoothing, is a non-parametric regression method that combines multiple regression models in a k-nearest-neighbor-based meta model to create a smooth line through a time plot or scatter plot to help visualize the relationships between variables. We used the non-parametric LOESS regression to fit a curve to the data (66). To prevent overfitting in estimating bandwidth, we used 10-fold cross-validation. We generated our predictive model using the data in the training set, and then measured the accuracy of the model using the data in the test set. We tested a range of bandwidths from 0.01 to 0.99 with a 0.01 step. The bandwidth resulting in the least sum of squares error was then selected (67).

### Neurosynth Decoding for Hubs Word Cloud Generation

Neurosynth (https://neurosynth.org/) is a platform for large-scale, automated synthesis of functional magnetic resonance imaging (fMRI) data. It uses information from several thousand published studies, reporting the results of fMRI studies, to determine the statistical association between cortical areas, and cognitive, disease, or function terms. Thus, every cortical vertex is assigned a statistical score of how correlated it is with terms within Neurosynth, and *vice versa*—every term in Neurosynth has a ranked by strength of an association list of cortical vertices associated with this term. This makes it possible to assess functions or disorders associated with a particular anatomical region in the cortex, with much greater statistical reliability than would be possible *via* visual inspection, for instance.

For Neurosynth decoding, surface maps showing all the hubs that exhibited significant age-dependent changes in the betweenness centrality metric (correlation between age and the betweenness centrality of nodes) in either the beta or gamma band-mediated networks were transformed using FreeSurfer from the surface to volume MNI space (mri_surf2vol). The correlation maps were then run through the Neurosynth decoding python module for the identification of the relevant text terms.

The text data significantly associated with the brain regions can be visually represented using word clouds (also known as text clouds or tag clouds); the more a specific word appears in a source of textual data, the bigger and bolder it appears in the word cloud. The Word cloud was generated using the first 500 most relevant terms from a total of 2,911 terms generated from the Neurosynth decoding module. The size of the words (Neurosynth terms) corresponds to its relative correlation with the maps as inferred by the Neurosynth decoding module. A Python package entitled “a little word cloud generator” was used for plotting the word cloud (https://github.com/amueller/word_cloud). Note that these word clouds are inherently statistical quantities, since only significant age-dependent changes were fed to the Neurosynth decoding module, and only significant correlations were included as part of the word clouds.

### Maturation of Resilience, Tested Using Neurosynth-Derived Hubs

To test the extent to which hubs identified in our primary analysis overlap with hubs that correspond to specific functions, we began by choosing 12 brain-function terms and extracting from Neurosynth the first 90 nodes in descending order of size, which corresponded to these terms. The sensory and cognitive terms were chosen because they are all known to mature between childhood and adulthood and represent a variety of cognitive functions that are known to rely more heavily on bottom-up or top-down processing. The DSM-5 terms were chosen because all the disorders with the exception of autism are likely to have an onset time in adolescence or early adulthood. Autism was added due to its high prevalence and our prior experience with the disorder, as well as due to the fact that the severity of autism sometimes increases during adolescence (68, 69). Note that we excluded psychosis and schizophrenia despite the high prevalence of the onset during adolescence. This is because these terms were not associated with any “reverse inference” maps in Neurosynth, i.e., there is no selectivity for which regions activate with these terms, hence making them non-specific for target hubs. The terms were entered exactly as they appear in the results section, except for the “dorsal visual” term, which was not available on the Neurosynth website. The term “dorsal visual” was generated to mirror the term “ventral visual,” using the neurosynth python framework (github.com/neurosynth), by specifying expression = “dorsal and visual,” in the dataset.get_studies module.

Reverse inference maps from Neurosynth (27) were downloaded for each of the examined terms at FDR = 0.05, as listed in the results section. The resultant meta-analytic reverse inference map, also known as the association test map, is a map of z-scores from a two-way ANOVA, testing for the presence of a non-zero association between the term(s) used and the voxels activation map. These maps were then projected and registered onto the FsAverage surface using pysurfer (pysurfer.github.io). The mean Z-scores from this two-way ANOVA, averaged across node's vertices for each of the 448 nodes, were then computed from these surface-projected maps. This mean z-score is shown as a textured color map on the cortex. Nodes were then removed from the graphs in order of their Neurosynth Z-scores, in descending order, from the highest z-score (i.e., the largest most important node) downwards. At each removal, the following two steps were performed: first, global efficiency for each subject was recalculated and normalized with respect to the original global efficiency before removal. The result at point M was the network resilience after the removal of M nodes. Then, the resultant-normalized global efficiency was correlated with age using Spearman correlation. The resultant correlations were then corrected using maximum statistics by permutation across bands (2 bands—beta and gamma), nodes removed (90 most connected, i.e., largest nodes), and terms (the 12 chosen from the Neurosynth database) using the methods described in the previous section. The resultant correlation is plotted at the maximum correlation. The correlation value for each node removal is shown as a color map on the top of the correlation plot, marked with the white line at which LOESS regression was plotted.

## Results

### Neurosynth Decoding for Hubs Word Cloud Generation

**Figure 2:**
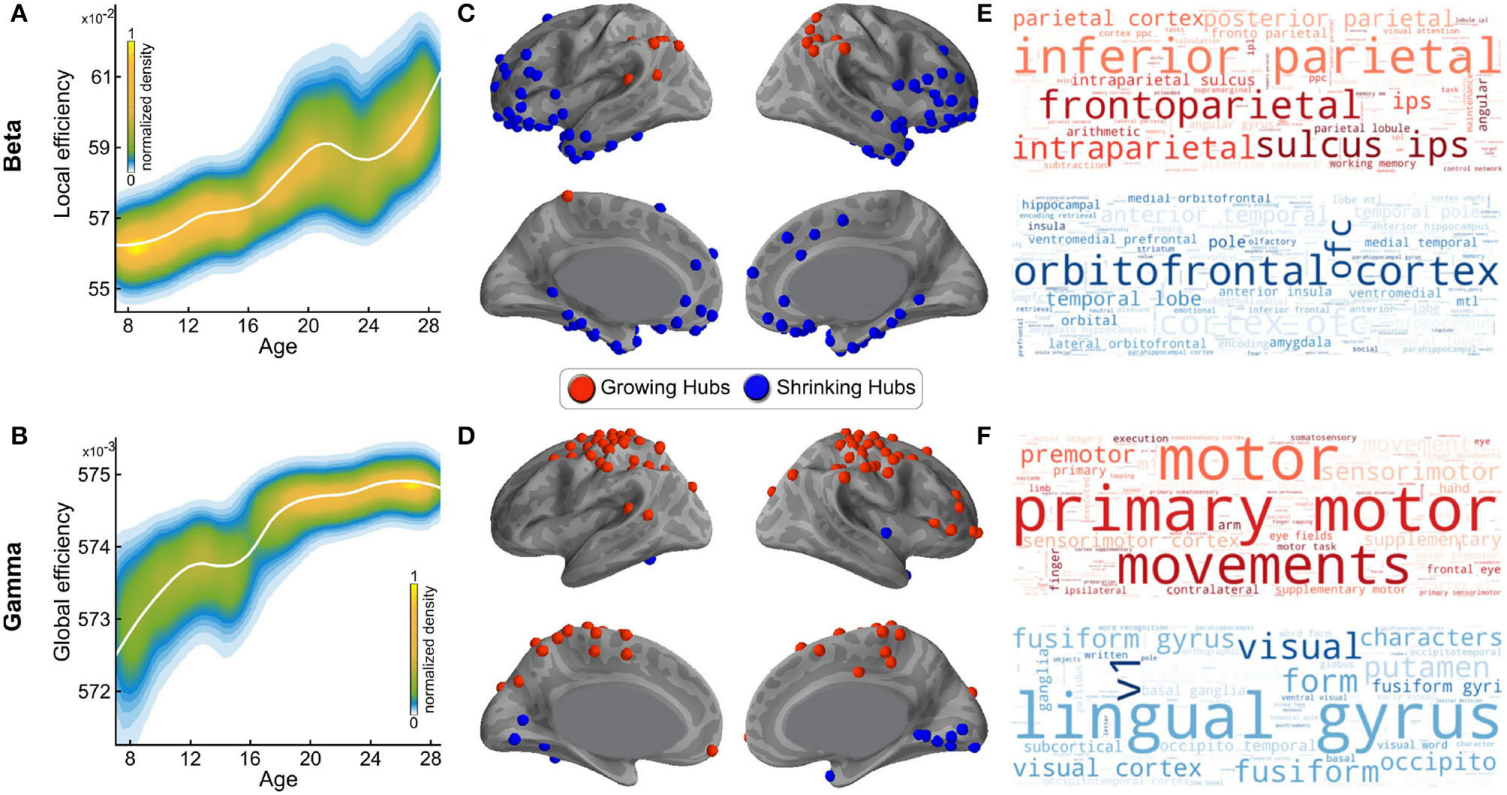
Spatial distributions of growing and shrinking hubs and their associated word clouds. **(A)** The LOESS plot (the solid white line) for the relationship between age and local network efficiency of beta band-mediated networks. The individual data points are represented using a normalized density color map, where each data point corresponds to one realization of the bootstrap procedure. **(B)** Like **(A)**, for gamma band-mediated networks, and global efficiency instead of local efficiency. **(C)** The spatial distribution of growing and shrinking hubs in the beta band-mediated networks. **(D)** Like **(C)**, for the gamma band-mediated networks. **(E)** Word clouds generated using wordle for the first 100 terms from Neurosynth for the beta band network- growing and -shrinking hubs. Larger font size reflects higher probabilistic association. The top (red) cloud was generated using the growing hubs, and the bottom (blue) cloud was generated using the shrinking hubs. **(F)** Like **(E)**, for the gamma band-mediated networks. **(A–D)** have been adopted from Khan et al. (22).

As noted in the introduction, in a prior study of resting state networks, we assessed the developmental trajectory of the graph theoretic metrics of local and global efficiency from age 7 to age 29 by frequency band (22). Specifically, we tested the maturation of these two graph theoretic efficiency metrics for each of the 5 intrinsic cortical rhythms—delta (1–4 Hz), theta (4–8 Hz), alpha (8–12 Hz), beta (13–30 Hz), and gamma (31–80 Hz). We found no significant age-dependent differences for either of these metrics in the three slower frequency bands (delta, theta, and alpha). In contrast, we found significant age dependence of network efficiency in both the beta and gamma frequency bands. More specifically, we found that resting state networks mediated by the beta brain rhythm become more locally efficient with maturation, i.e., more organized into clusters and connected with nearby regions (Figure 2A), while networks mediated by the gamma brain rhythm become more globally efficient with maturation, i.e., prioritizing faster signal transmission between distant cortical regions (Figure 2B). In the same prior study, we used the betweenness centrality graph metric to identify which of the hubs associated with each of the two networks changed significantly in efficiency with age. Two categories of hubs emerged from this analysis: hubs that grew—i.e., gained nodes—with maturation, and hubs that shrunk—i.e., lost nodes—with maturation. The distribution of the hubs that grew or shrunk significantly with age in the beta band network is shown in Figure 2C, and the distribution of the hubs that grew or shrunk significantly with age in the gamma band network is shown in Figure 2D.

In order to test our hypothesis regarding the functional roles of the hubs found to grow or shrink with maturation within each of the two identified networks (in Figures 2C,D), and thus gain a better understanding of their functional significance, we then tested which Neurosynth terms were most associated with these hubs, for networks mediated by either the beta or gamma bands. To that end, we extracted from Neurosynth the list of terms associated with each of the regions marked in Figures 2C,D, as ranked in order of their relevance for that region, as ranked by Neurosynth. The statistically generated word cloud associated with these hubs is shown in Figure 2E for beta band-mediated networks and, in Figure 2F, for gamma band-mediated networks. The word clouds within each panel are further broken down by whether the hubs are growing with maturation (red), or shrinking with maturation (blue), signifying greater or reduced reliance on these hubs with maturation, respectively. The larger text corresponds to a higher combined statistical rank within Neurosynth across the corresponding regions (growing/shrinking hubs).

### Maturation of Resilience, Tested Using Neurosynth-Derived Hubs

**Figure 3:**
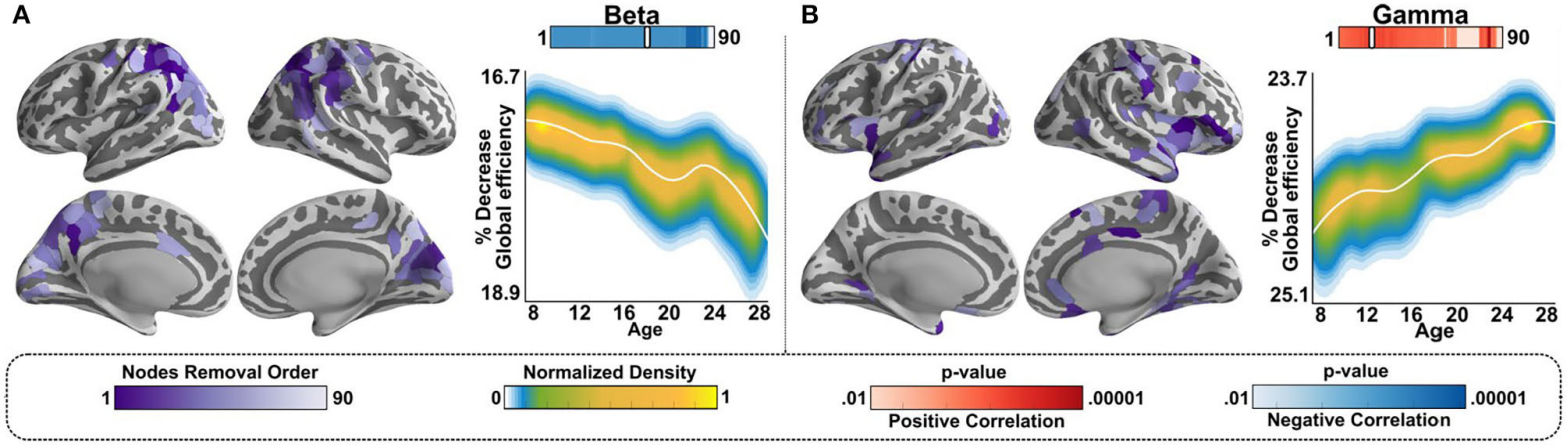
Resilience in beta and gamma-mediated networks follows opposite developmental trajectories. **(A)**-Left panel: Hubs identified from the beta band network (Figure 2C) were first transformed to Neurosynth coordinates then and ranked by size. Approximately, 448 nodes in total were identified, and the order of node removal for testing resilience, by node size, is color coded on the purple color bar, thresholded to avoid saturation. **(A)**-Right panel: After each removal of a node, in ranked order, we evaluated whether there was a significant effect of age generated by the removal of that node. Shown is the LOESS plot for one instance of the effect of age at 54% of nodes removed, where significance of age effect was maximal. The individual data points are represented using a normalized density color map, where each data point corresponds to one realization of the bootstrap procedure. The top bar, labeled “Beta,” shows how significant the effect of age was at any number of nodes removed, where significance is color-coded using the “*p*-value” color bars on the bottom (red for relatively increased resilience with age, blue for relatively decreased resilience with age). The white notch on the bar under “Beta” is the snapshot, i.e., number of nodes removed at that point; we chose to plot to show the weakening of resilience with age. **(B)-**Left panel: Same as **(A)**-left panel for the gamma band-mediated networks, using the hubs identified for the gamma band-mediated network (Figure 2D). **(B)**-Right panel: Same as **(A)**-right panel for the gamma band-mediated networks.

Network resilience is a metric that assesses the relative significance of a hub for maintaining the network's capacity to integrate information by removing hubs from the network, from largest to smallest in descending order and evaluating network efficiency relative to the number of nodes removed. Because resilience is evaluated using hubs, it is very well-suited to assess the potential functions of hubs. We have previously shown that resilience in beta band-mediated networks decreased with age, while resilience in gamma band-mediated networks increased with age, as illustrated in Figure 3 (22).

**Figure 4:**
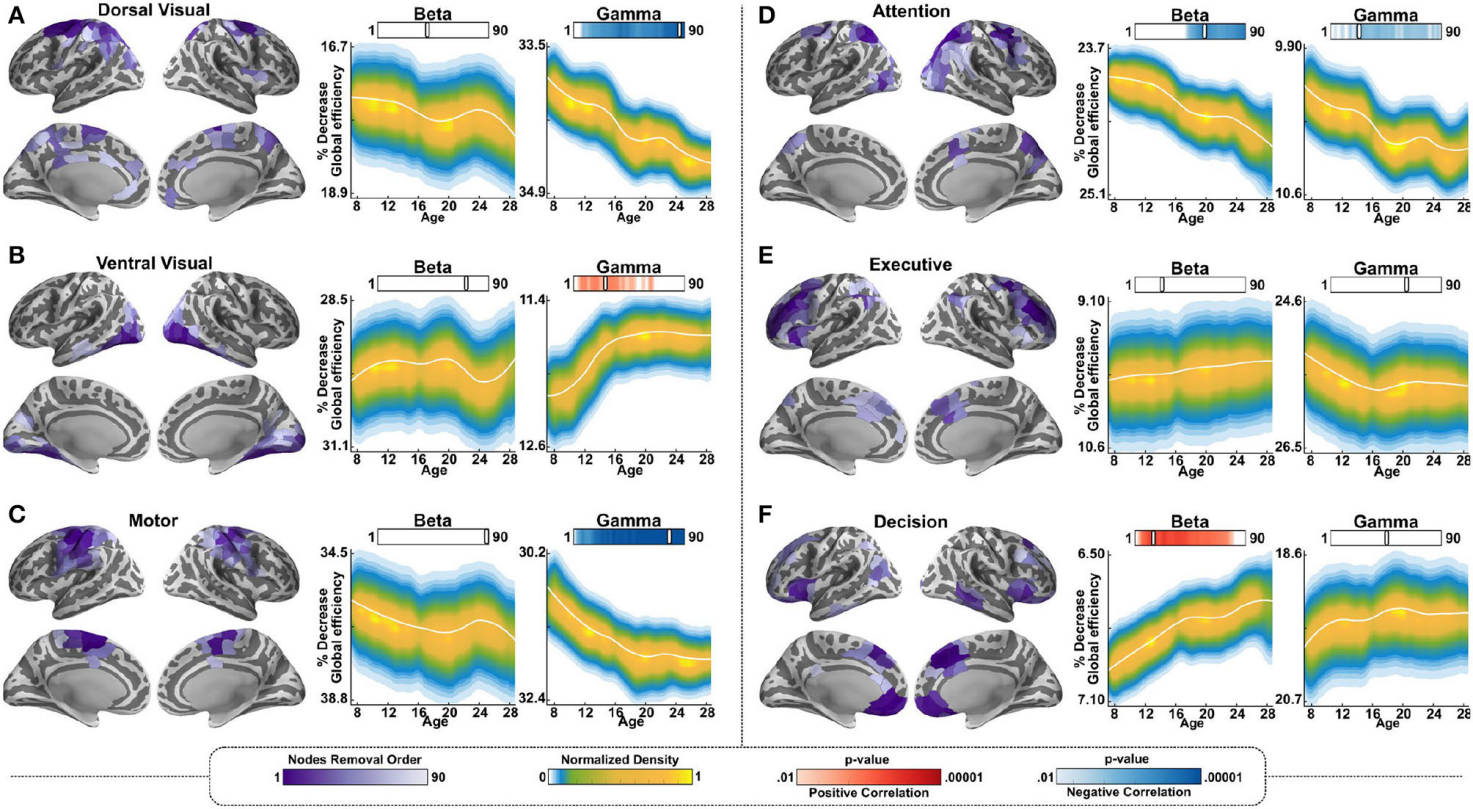
Resilience in gamma and beta-mediated networks for relative networks associated with sensory and cognitive terms. This analysis replicated the process shown in Figure 3, but instead of using the actual hubs of each network, it used the largest 90 hubs determined by Neurosynth to be associated with the term being tested in descending order of size. The same hubs were used for both the beta and gamma-mediated networks. **(A)** Left side (subtitled with the relevant term): a representation on the cortex of the cortical Neurosynth nodes associated with the term “Dorsal Visual,” with a rank indicated by the purple color bar at the bottom, and thresholded as in Figures 3A,B. The middle panel (subtitled “Beta”): Resulting resilience for the beta band network for one “snapshot” of percentage of nodes removed, marked by the notch in the bar under the word “Beta.” The right panel (subtitled “Gamma”): Same as the middle panel, but for the gamma band-mediated network. **(B)** Same as **(A)** for the term “Ventral Visual.” **(C)** Same as **(A)** for the term “Motor.” **(D)** Same as **(A)** for the term “Attention.” **(E)** Same as **(A)** for the term “Executive.” **(F)** Same as **(A)** for the term “Decision.” *P*-value color bars: red indicates resilience increased significantly with age, and blue indicates resilience decreased significantly with age.

To further investigate the functional roles of the mapped maturing hubs, we statistically mapped and identified the hubs associated with specific meta-analytic terms and then tested whether and how their removal from the network affected the resilience of each of the two networks. To that end, 12 Neurosynth terms were chosen, with a focus on terms that could help in differentiating bottom-up functions from top-down functions. We began by selecting three terms associated with basic visual or motor functions and, therefore, bottom-up processes: “dorsal visual” stream, “ventral visual” stream, and “motor system.” We hypothesized that these sensory-centered networks are more strongly dependent on feedforward connectivity, and, therefore, should show greater age-dependent impacts in the gamma band. Indeed, we found that, for all these terms, removing their associated hubs resulted in no significant beta-mediated age effects. However, the removal of these same hubs resulted in highly significant differences in the gamma band-mediated age-dependent network resilience. More specifically, for both the dorsal visual stream and the motor system, removal of the associated hubs resulted in significantly age-dependent resilience, with greater resilience (i.e., relatively less decrease in global efficiency) in children relative to adults in the gamma band. In contrast, removal of the hubs associated with the ventral visual stream resulted in significantly age-dependent resilience in the opposite direction in the gamma band, with adults showing a significantly reduced impact on global efficiency with removal of the hubs relative to children (Figures 4A–C).

We then repeated the same analysis with three terms associated with cognitive functions known to be mediated by top-down processes: “Attention,” “Executive” (for executive function), and “Decision.” We hypothesized that networks associated with these terms are more strongly dependent on feedback connectivity and, therefore, should show greater age-dependent impacts in the beta band-mediated networks. Networks associated with these processes are also known to mature substantially during adolescence. Contrary to our hypothesis, the results for this group of cognitive terms were mixed. Using the hubs from the attention network to test resilience resulted in reduced resilience with age in both the beta and gamma bands. This means that the younger age groups were less severely impacted by the removal of the hubs than the older age groups. Using the hubs from the executive function network resulted in no effect of age and using the hubs from the decision network resulted in age-dependent resilience in the beta band only, with the older age group being less impacted than the younger age group (Figures 4D–F).

**Figure 5:**
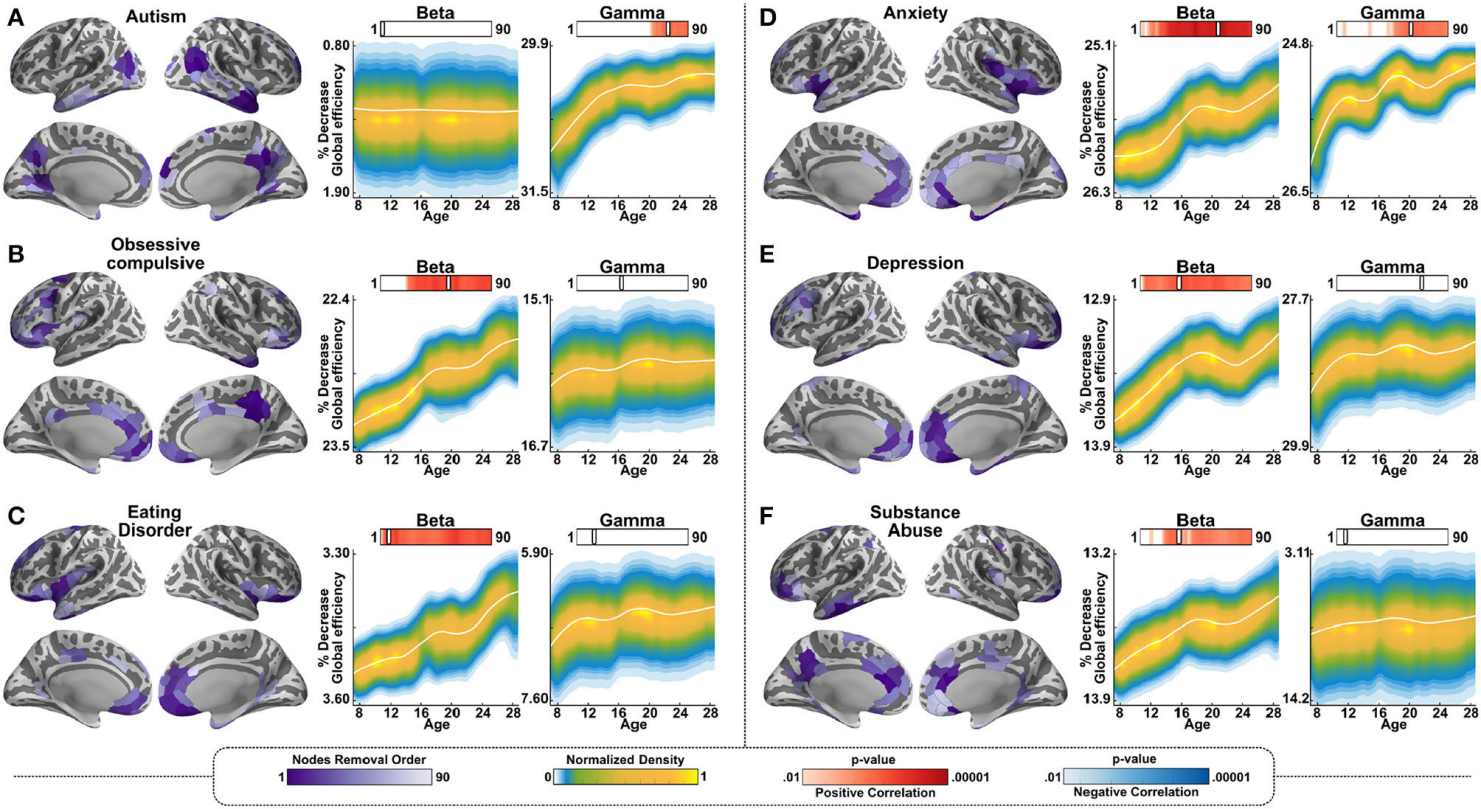
Resilience in gamma and beta-mediated networks for relative to networks associated with psychiatric terms. The same as Figure 4, but for the following 6 terms: **(A)** Autism. **(B)** Obsessive Compulsive. **(C)** Eating Disorder. **(D)** Anxiety. **(E)** Depression. **(F)** Substance Abuse.

Lastly, we tested resilience using the hubs associated with DSM-5 disorders that are common in adolescence. This part of the analysis was data driven rather than hypothesis driven, and the aim was to test whether resilience changes associated with each of these terms manifest differentially in the networks mediated by the beta vs. the gamma bands. Specifically, we tested the changes in the resilience of the networks, with the removal of the hubs associated with the following terms: “Autism,” “Obsessive-Compulsive,” “Eating Disorder,” “Anxiety,” Depression,” and “Substance Abuse.” Removal of the hubs associated with all of these disorders, with the exception of autism, resulted in increased resilience in the older participants relative to the younger participants in the beta band, similarly to the cognitive decision network. In other words, the removal of the hubs resulted in less decrease in global efficiency for the older age group relative to the younger age group. In the gamma band, an age-dependent change in resilience was observed for the terms “autism” and “anxiety”; for both of these terms, in the gamma band, resilience was significantly more impacted in the younger age groups than in the older ones by the removal of the hubs (Figures 5A–F).

## Discussion

This study aimed to test the hypothesis that maturing (growing or shrinking) hubs associated with resting state networks mediated by the beta frequency band are more likely to be associated with top-down processing while maturing hubs associated with resting state networks mediated by the gamma frequency band are more likely to be associated with bottom-up processing. The results showed that the hubs that we have previously shown to change during maturation in the gamma band-mediated network, which increased in global efficiency with age, were more likely to be statistically associated with sensory and motor terms in Neurosynth, and thus more likely to be associated with feedforward, i.e., bottom-up processes. In contrast, the hubs that we have previously shown to change during maturation in the beta band-mediated network, which increased in local efficiency with age, were more likely to be statistically associated with more terms in Neurosynth that reflect more complex cognitive function, and thus more likely to be associated with feedback, i.e., top-down processes. These findings support the hypothesis that intra-areal beta rhythm synchronizations preferentially mediate top-down functions, while intra-areal gamma rhythm synchronizations preferentially mediate bottom-up functions.

These emergent patterns are consistent with the literature in the field, showing a preferential role for the gamma band in mediating bottom-up processes, even if not exclusively so, and a preferential role for the beta band in mediating top-down processes, even if not exclusively so. More specifically, the pattern of shrinking the frontal hubs observed in the beta band is consistent with studies showing reduced frontal task-related activation with maturation, for instance, for inhibitory control, potentially due to increased efficiency of top-down communication, putatively mediated by the beta band (25). In line with this, the top Neurosynth terms emerging from our decoding analysis for the beta band hubs were “orbitofrontal” (shrinking hubs), “frontoparietal,” and “inferior parietal” (growing hubs). These regions are associated with processes that are generally considered to be top-down, such as attentional control (70), executive control (71), and decision-making. Gamma-mediated networks showed an increase in global efficiency with maturation, which is consistent with the putative role of gamma for mediating bottom-up connectivity (17, 72) as new connections would have to be formed to carry information forward to developing frontal brain regions. Indeed, two of the top Neurosynth terms emerging from our decoding analysis for the gamma band hubs that were shrinking or growing with maturation were “primary motor” (growing) and “lingual gyrus” (shrinking). The observation that the motor, dorsal visual, and ventral visual systems showed age-dependent resilience only in the gamma band may reflect the fact that all of these processes rely heavily on feedforward inputs. In other words, these regions are associated with processes that are generally considered to be bottom-up, such as the generation of motor movements (primary motor), and the processing of visual inputs (lingual gyrus). Indeed, MEG in humans (73) and non-human primate studies (72, 74, 75) demonstrate that gamma rhythms flow up in a bottom-up direction, spreading from lower-order visual sensory regions to higher-order regions, while Beta rhythms flow in a top-down direction, spreading from higher-order multimodal regions to lower-order sensory regions.

The mixed results in the cognitive domain terms we tested for overlap in the resilience-based analysis likely reflect the far more complex processing and complex networks associated with the chosen terms—attention, executive function, and decision. The attention network is, indeed, known to be mediated heavily by both feedforward and feedback inputs in line with our results and reflects the significance of both beta and gamma to higher-order functions. The executive function network showed no age effect likely because, unlike the other terms, there were no hubs associated with it in the reverse influence analysis, which may reflect that the meta-analytics maps associated with it in Neurosynth's ranking are ambiguously defined. In contrast, the decision network clearly relies most heavily on feedback connectivity and, indeed, showed age-dependent resilience only in the beta band. The differentiation between the three analyzed groups of terms confirms that the presented results, indeed, have implications for cognitive function.

The pattern observed for terms describing psychiatric disorders using this analysis also suggests that the mediating frequency bands have specific and differential roles. Notably, five of the six chosen disorder terms are more commonly associated with the later onset (adolescence to early adulthood) and showed age-dependent resilience in the beta band. In contrast, the only disorder on the list that is considered neurodevelopmental, i.e., early origin, autism, showed age-dependent resilience only in the gamma band. This suggests that beta band networks might not undergo normal development in autism during adolescence, and thus are particularly or more severely impacted in line with prior findings (12, 22, 76, 77). Anxiety was the only term that corresponded to networks showing age-dependent trajectories in both the beta and gamma bands differentiating it from the other tested terms. It is possible that these anxiety networks are relatively poorly defined when anxiety type is not specified, and, thus, multiple networks are captured by this term in Neurosynth. Indeed, there are many subtypes of anxiety disorders that were not differentiated in our Neurosynth search (e.g., social anxiety, performance anxiety, generalized anxiety, etc.). While the other disorders tested are typically associated with specific onset times windows (e.g., childhood for autism, adolescence for eating disorders or depression), anxiety can arise at any age, and, therefore, a maturation trajectory for its corresponding networks may not be as well-defined as it is for the other disorder terms tested.

A potential limitation of this paper is that it sought to build on prior results to further refine our understanding of these results. Because the prior results only showed age-dependent changes in the beta and gamma band-mediated networks, we only focused on these two networks here too and did not examine the hubs associated with the delta, theta, and alpha bands. It is possible that specific hubs within those networks do show age-dependent differences even if the network as a whole does not. It is also possible that age-dependent bandwidth changes within specific bands, which were not considered in the prior study, might have an impact on maturation trajectories in the slower frequency bands in particular. Future studies are needed to further elucidate these questions.

This study added meta-analytic tools to our prior study of frequency-specific maturation of resting state networks. The goal of these additional analyses was to assess the potential functional significance of the hubs identified in the prior study. While it is clear that both beta band and gamma band-mediated resting state network networks are highly complex and contribute to processing in a multitude of ways that are not necessarily or exclusively direction specific, the Neurosynth-derived results are, overall, consistent with our prior hypotheses that beta band-mediated networks are likely to be more heavily weighted toward top-down processing, while gamma band-mediated networks are likely to be more heavily weighted toward bottom-up processing. Mechanistically, both of these cortical rhythms are mediated by GABAergic systems (15, 78); the maturation of GABAergic processes extends well into adolescence and early adulthood (79), and the maturation of GABAergic systems likely also underlie the maturation of these cortical networks, and thus hub topology. Thus, the maturation of GABAergic systems is highly likely to influence the maturation of both networks and mediates developmental changes in both bottom-up and top-down processing. Lastly, this study demonstrates that Neurosynth can be employed to investigate the functional role of networks and their hubs, even in the absence of direct functional data.

## Data Availability Statement

The raw data available for sharing will be provided by the authors following the approval of the required Massachusetts General Hospital Sharing Agreement. Requests should be directed to the corresponding author(s).

## Ethics Statement

The studies involving human participants were reviewed and approved by Massachusetts General Hospital IRB Board. Written informed consent to participate in this study was provided by the participants' legal guardian/next of kin for minors, and by the participants themselves for adults.

## Author Contributions

SK, JAH, FM, MSH, and TK designed the study. SK, JAH, and FM analyzed the data. SK, JAH, FM, and TK wrote the paper. All authors contributed to the article and approved the submitted version.

## Funding

This work was supported by grants from the Simons Foundation (SFARI 239395; TK), the National Institute of Child Health and Development (R01HD073254; TK), the National Institute of Mental Health (R01MH117998 and R21MH116517; TK), the National Institute for Biomedical Imaging and Bioengineering (P41EB01589 and P41EB030006; MSH), and the National Institute of Neurological Disorders and Stroke (R01NS104585; MSH).

## Conflict of Interest

The authors declare that the research was conducted in the absence of any commercial or financial relationships that could be construed as a potential conflict of interest.

## Publisher's Note

All claims expressed in this article are solely those of the authors and do not necessarily represent those of their affiliated organizations, or those of the publisher, the editors and the reviewers. Any product that may be evaluated in this article, or claim that may be made by its manufacturer, is not guaranteed or endorsed by the publisher.
